# Electrocortical activity during resistance exercises in healthy young adults—a systematic review

**DOI:** 10.3389/fspor.2024.1466776

**Published:** 2024-11-27

**Authors:** Anton Visser, Daghan Piskin, Daniel Büchel, Jochen Baumeister

**Affiliations:** Exercise Science and Neuroscience Unit, Department Exercise and Health, Paderborn University, Paderborn, Germany

**Keywords:** neurophysiology, brain, EEG - electroencephalography, strength training, exercise load, volume, type of muscle contraction

## Abstract

**Introduction:**

Resistance training (RT) is known to induce both peripheral and central adaptations, resulting in enhanced strength, sports performance, and health benefits. These adaptations are specific to the training stimuli. The acute cortical mechanisms of single sessions resistance exercise (RE) are not yet understood. Therefore, this review investigates the electrocortical activity during acute RE regarding the specific RE stimuli.

**Methods:**

A systematic literature search was conducted across three databases, focusing on the acute electrocortical activity associated with the muscle contraction type, load, and volume of RE in healthy young adults.

**Results:**

Out of an initial 1,332 hits, 19 studies were included for data synthesis. The findings from these studies show that the RE load, contraction type, and volume during RE significantly affect brain activity. The current literature exhibits methodological heterogeneity attributed to variations in study quality, differences in the location of cortical sources, the cortical outcome parameter and the use of diverse training interventions.

**Discussion:**

Despite inconsistencies in the current literature, this review highlights the need to investigate time and frequency-specific characteristics when examining electrocortical activity during RE. More research is necessary to further explore the acute cortical mechanisms related to resistance exercise. Future research could improve our understanding of acute neural responses to RE and provide insights into mechanism underlying more long-term neuroplastic adaptations to RT.

## 1 Introduction

Resistance exercise (RE) is a planned and structured form of physical activity (1), aiming to improve muscular fitness by performing muscle contractions against external resistance When performing RE in a repeated and regular manner, referred to as resistance training [RT; (1, 2)], long-term adaptations of neuromuscular function can be observed. Therefore, RT is known to induce health and performance benefits, with the most prominent adaptation being the maintenance or gain of strength (3). An increase in strength induced by RT was demonstrated in healthy adults (4), older adults (3, 5), rehabilitation (6), and athletes (7, 8). Interestingly, these long-term adaptations to RT have been demonstrated not only in the peripheral muscular system but also in the central nervous system, particularly the brain (9–12). Therefore, these structural and functional changes in the brain coming along with RT are supposed to support neuromuscular performance due to an improved recruitment of motor units from brain motor areas (13–15).

The physiological adaptations to RT result from the specific stimuli induced throughout regular and repeated bouts of RE (4). Therefore, the exercise variables prescribed for the single RE bouts within RT are decisive for the short- and long-term functional adaptations to RT, including those observed in the brain (16). Here, the two main exercise variables that determine effort during a single bout of RE are exercise load and exercise volume (17). For the single bout of RE, load is typically quantified by the weight lifted by the individual. It is a key component to quantify the acute external load (the physical work performed) during an acute bout of RE, because it defines the neuromuscular strategies such as the motor unit recruitment (18). In addition to the load, the total number of repetitions performed during a training session needs to be considered. The RE volume is defined as the product of repetitions and the resistance load prescribed (4, 19) and influences recruitment strategies which aim to counteract the manifestation of neuromuscular fatigue (20). Both exercise volume and load are critical factors in determining the RE stimuli that promote neuromuscular adaptations, because they impact the global energetic and neural recruitment demands associated with RE (17, 19, 21, 22). In addition, RE variables such as rest intervals can be utilized to control the load and volume of the exercise session (23, 24). Additionally, the type of muscle contraction - categorized as fixed muscle fiber length [static; (25)], shortening of muscle fibers [concentric; (26)], or lengthening of muscle fibers [eccentric; (26)] - influences strength gain in RE. Research has revealed variations in anabolic responses (27), motor unit activation (28, 29), and brain activity (29) across these contraction types. Consequently, each muscle contraction type involves unique neuromuscular mechanisms, leading to specific stimuli and adaptive responses.

Taken together, load, volume, and type of muscle contraction affect muscle adaptations and strength (30). The manipulation of RE variables and its consequences for energetic and neural recruitment strategies are therefore may affect the functional changes in the brain following RE (31). Therefore, understanding how the manipulation of these training variables affects (neuro-) physiological responses is fundamental for designing and prescribing targeted training stimuli (30).

The current literature on neural mechanisms underlying RE reveals challenges in gaining insights into the brain's contribution to RT (32, 33). As discussed above, the complex interaction of RE variables, including load, volume, and type of muscle contraction, makes it challenging to gain systematic insights into the braińs contribution to strength. Additionally, the methodological heterogeneity of neuroscientific approaches complicates the systematic investigation of the neural mechanisms of RT, because different neuroscientific methods describe neural underpinnings on varying scales of temporal and spatial precision (34). Thus, the interaction between RE and acute adaptive responses in the structure and function of the brain is not yet well examined (35).

Despite the challenges in examining neural activation in RE due to variations in RE regimes and methodological approaches, electroencephalography (EGG) is a highly recommended method for depicting acute neural mechanisms underlying RE (36, 37). Due to excellent temporal resolution and a high degree of mobility, EEG has been used to depict changes in brain activation associated with the intensity (38, 39) and fatigue (40) of aerobic exercise. Furthermore, EEG has been useful in characterizing the motor state in smaller resistive tasks such as handgrip, and in illustrating fatigue in handgrip (41, 42) and knee movement (43, 44). Additionally, EEG potentially is a reliable tool for measuring cortical activity during RE, as excellent reproducibility in measuring the degree of brain activation during moderate loads of RE has been reported across frequency bands ranging from 2 to 30 Hz (45). Unlike magnetoencephalography (MEG) and functional magnetic resonance imaging (fMRI), which have limited mobility, and functional near-infrared spectroscopy (fNIRS), which has limited temporal resolution (34), EEG provides real-time, direct measurements of the fast cortical processes contributing to RE. Therefore, the high temporal resolution in mobile settings is crucial for understanding immediate brain activity to RE. Nevertheless, some methodological considerations must be considered when recording EEG during RE. While the EEG's spatial resolution seems to be sufficient to differentiate between resistance knee and ankle exercises (46), the EEG has a lower spatial resolution compared to fMRI, MEG, and fNIRS (34). Further, since the EEG records a sum signal of electrical potential changes in the electrode subspace, recordings are prone to contamination from intense muscle activity (47). Fortunately, advanced techniques such as independent component analysis (ICA) can effectively distinguish between cortical and non-cortical contributions to the EEG signal and allow for analysis of brain activity during intense muscle contractions (48).

The primary aim of this systematic review is to provide an overview of the electrocortical activation underlying acute single RE session in healthy young participants. The review focuses on exercise variables in acute RE that potentially impact the brain, including load, volume, and type of muscle contraction. Therefore, the specific aims of this research are to (i) synthesize the existing evidence on the cortical activity underlying acute bouts of RE with regards to the variables load, type of muscle contraction, and volume and (ii) to assess the methodological quality the current literature investigating the cortical activity underlying acute bouts of RE. The study findings should provide prospects for future EEG-based studies on brain function during RE.

## 2 Methods

The objectives of this study, with respect to the PICO (49) scheme, were to provide an overview of the electrocortical activation (Outcome) underlying RE variables (Intervention) in healthy young adults (Participants). Here, healthy young adults are defined as individuals aged 18–35 years without chronic diseases, current injuries, or medications that could significantly impact physical function or cortical activity. The outcome parameter must be an indicator of electrocortical activation derived from EEG and compared to a control condition (Comparison). The control condition could be represented by a resting situation or another clearly distinguished intervention, such as RE with different types, volumes, or load. Accordingly, criteria to check the eligibility of studies are defined as referring to (1) an investigation of healthy young adults, (2) an intervention performing an acute bout of RE, (3) reporting an EEG outcome parameter recorded during an acute bout of RE, (4) comparing different types, loads or volume of RE. Studies were excluded, if they were non-English publications, non-peer or limited review conference proceedings, book chapters, or reported only diseased population groups. Further studies were excluded which exclusively investigate finger, wrist or feet movements. Studies were included if at least one intervention group met all eligibility criteria. So, control groups of studies investigating diseased or older adults were also considered for potential inclusion [for example (50, 51)].

A systematic literature search strategy was performed on the 11th of March 2024 in the databases Scopus, Web of Science, and Pubmed. The search term was generated by controlling a combination of terminologies, including “EEG”, “resistance exercise”, and at least one of the terms related to the volume or load of exercise or type of muscle contraction. The following keywords were used in the three databases: (1) “EEG”, “Electroencephalography” and (2) “force”, “resistance”, “strength*”, “weight”, “power” and (3) “exercise”, “physical activity”, “muscle activation”, “muscle activity”, “muscle contraction”, “voluntary contraction” and (4) “(Isotonic AND Isometric)”, “(Isotonic AND Isokinetic)”, “(Isometric AND Isokinetic)”, “(Concentric AND Eccentric)”, “(Concentric AND Isometric)”, “(Eccentric AND Isometric)” or (5) “intensity”, “force level”, “torque level”, “velocity”, “tempo”, “speed” or (6) “training load”, “repetition*”, “set”, “sets”. The terms were connected with “OR” within each of the six combination groups, and the combinations 1–3 were combined using “AND” and concatenated with one of the groups 4–6 using “OR”.

In the database Scopus, the search was performed in the fields of article title, abstract, and keywords. In Web of Science and Pubmed the search was performed in all fields. All identified articles were independently screened by two authors (AV & DP). Duplicate references were automatically removed the screening process was conducted using Citavi (Version 6.15, Swiss Academic Software GmbH, Switzerland). Both reviewers needed to completely agree on the eligibility of a study for it to be included. In case of disagreement between the authors AV and DP regarding study eligibility, all authors collectively contributed to resolving the discrepancy to arrive at a unanimous decision. Data extraction focused on population characteristics, intervention details, EEG outcome parameters, cortical areas examined, and EEG findings. The data was collected based on the written information provided in the included studies, without contacting authors for missing data. To address the aims of this review, we systematically synthesized the included studies to document: (i) cortical activity underlying RE with respect to exercise variables (load, contraction type, and volume), and (ii) the methodological quality of the current literature. The latter was assessed based on RE determinants [(30), see Table 4], methodological details of EEG (see Table 5), and a risk of bias assessment (Figure 2).

The quality of the included studies was evaluated using the Quality Assessment Tool for Observational Cohort and Cross-Sectional Studies from the National Heart, Lung, and Blood Institute (52). This tool includes 14 items that probe potential biases, confounding elements, study power, and the strength of causality between dependent and independent variables, among other factors. All included studies are cross-sectional studies in which cortical activity is recorded during RE. So that both exposure and outcome are measured once within the same timeframe. Therefore, items 10 and 13 were not considered in this assessment, as they refer to the repeated measurement of exposure and loss to follow-up, which are not applicable to the cross-sectional studies included. The evaluation of a population being free from the outcome of interest at the beginning is predominantly important in clinical settings and not applicable to cross-sectional studies including healthy adults. Therefore, item three has been excluded from the quality assessment. Quality assessment was independently carried out and reported by two reviewers (AV & DP). Each item on the tool was rated as either “no”, “not reported”, or “not applicable”, indicating a potential risk of bias, or “yes”, suggesting minimized bias risks. In case of disagreement between the two reviewers, both reviewers reached a consensus with the involvement of the other authors to arrive at a rating for each study on each item.

## 3 Results

**Figure 1:**
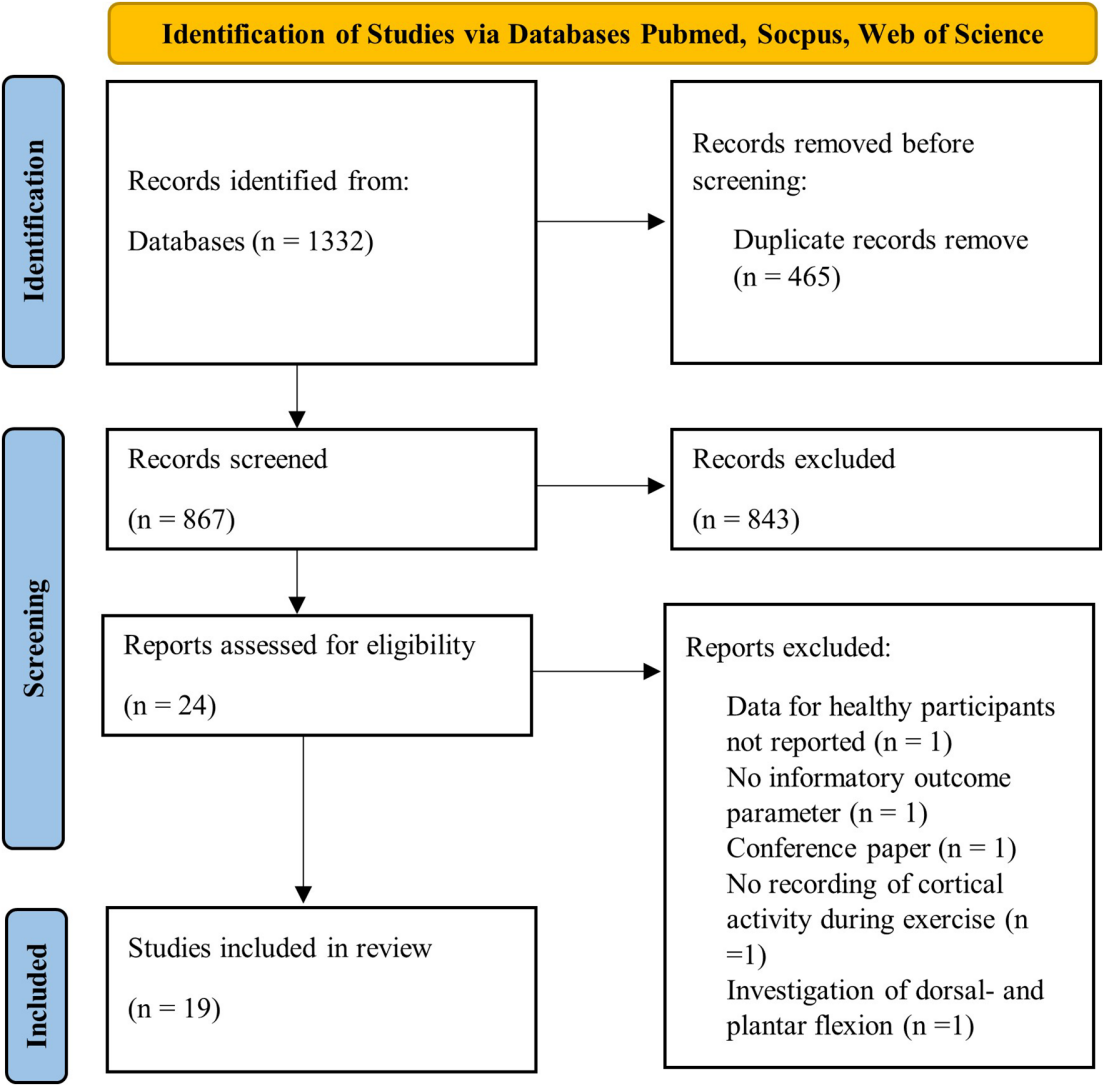
Flow diagram of study selection using PRISMA guidelines (53).

The initial systematic database search identified 1,332 records. Figure 1 illustrates the flow diagram of the study selection process. 465 duplicates were removed, and 843 publications were excluded in the process of screening titles and abstracts. 24 studies were screened as full-text assessments. Five of these publications were excluded due to missing outcome data for a healthy population (*n* = 1), no informatory outcome parameter (*n* = 1), conference paper (*n* = 1), absence of recorded cortical activity during exercise (*n* = 1), and their exclusive investigation of dorsal- and plantarflexion (*n* = 1). Eventually, 19 studies were included for the final data synthesis.

In sum the included studies investigated 252 participants (49f, 193m), with each group in the mean consisting of 12.6 (±5.3) participants. The population examined in this review consists of young, healthy adults under the age of 35, who are not suffering from any neurological diseases or injuries that would potentially impair RE or cortical activity. In three studies healthy people with expertise in specific sports like endurance or resistance training have been investigated (54–56). Out of 19 included studies, twelve investigated the RE variable load (Table 1), seven types of contraction (Table 2), and one volume (Table 3). Gwin & Ferris (46) is listed for both types of contraction and load of exercise, as both RE variables were examined and reported separately. Tables 4, 5 show the examined brain localizations in relation to the corresponding studies about load, type of muscle contraction, and volume in resistance training. Table 6 maps studies using channel-based brain localization, while Table 7 presents studies utilizing source-based localizations for each of the RE variables. The allocation of brain areas follows the classification provided by Doborjeh et al. 2020 [see Figure 5 (70)]. Four studies were not considered in these summary tables because of a limited assignment of their cortical outcome parameters to specific brain regions. This was the case for studies that examined corticomuscular coherence (CMC), thus investigating a mixed signal consisting of synchronization of central neural activity with muscle activity (50, 66). Additionally, it applies to studies that focused on topographical changes in brain activity based on connectivity patterns (60, 61).

**Table 1:** Electrocortical correlates and load of resistance exercise of the included studies.

Author	Population	Muscle/exercise	EEG parameter	Brain area	Cortical findings
(57) Abeln et al., 2013	*N* = 11 (7 m, 4f, 22–45 y)	Isometric right knee Ext (20, 40,60,80,100% MVC)	Cortical Current Density	PMC, M1, S1, SAC	CCD increased with leg extension intensity highest cortical activity in M1 ipsilateral cortical activity in M1 higher than contralateral
(50) Bayram et al., 2015	*N* = 20 (10f, 10 m, 22.60 ± 0.90)	Isometric left elbow Flex (20%, 50, 80% MVC)	CMC	Right sensorimotor cortex ROI	relationship between CMC and force force level effect of agonist (BB & BR) at C4 CMC antagonist (TB) no main force level effect
(58) Bayram et al., 2023	*N* = 20 (10f, 10 m, 22.60 ± 0.87)	Isometric left elbow Flex (20%, 50, 80% MVC)	Absolute and relative ESP	right primary sensorimotor ROI	Significant increase from 50 to 80% MVC in beta and low gamma No significant differences between 20, 50 and 80% MVC in Delta, Theta, Alpha, Beta and Low-Gamma absolute ESP Significant decrease of relative beta ESP between 20 and 50 and 50 to 80% MVC No significant differences between 20, 50 and 80% MVC in Delta, Theta, Alpha, and Low-Gamma relative ESP
(51) Cremoux et al., 2013	*N* = 8 m (28.14 ± 3.98)	Isometric right elbow Ext (25, 50, 75% MVC)	20 Hz ERD	C3	ERD increased with increasing force level in extension
(54) Dal Maso et al., 2012	*N* = 11 ST m (24.10 ± 4.31)	Isometric right knee Flex & Ext (20, 40, 60, 80% rMVC)	TRSP	Cz	suppression in 21–31 Hz frequencies with increasing force level in Flex No significant effect of Force level on 13–21, 35–45 Hz TRSP in Flex and Ext
	*N* = 10 ED m (22.09 ± 2.30)	Isometric right knee Flex & Ext (20, 40 60, 80% rMVC)	TRSP	Cz	No significant effect of Force level on 13–21,21–31, and 35–45 Hz TRSP in flexion and extension
(55) Dal Maso et al., 2017	*N* = 11 ST m (24.10 ± 4.31)	Isometric right knee Flex & Ext (20, 40 60, 80% rMVC)	CMC 13–21 & 21–31 Hz	Cz	Torque Level effect on CMC magnitude 13–21 Hz & 21–31 Hz in flexion and extension
	*N* = 10 ED m (22.09 ± 2.30)	Isometric right knee flexion & extension (20, 40 60, 80% rMVC)	CMC magnitude 13–21 Hz & 21–31 Hz	Cz	Torque Level effect on CMC magnitude 13–21 Hz & 21–31 Hz in flexion and extension
(59) Fry et al., 2014	*N* = 15 m (24 ± 5 y)	Isometric right knee extension 15, 30, 45, 60% MVT	Cortical Current Density	Sensorimotor cortex ROI	Gamma band cortical activity increased with contraction torque Other specific frequency bands were unaffected by torque
(46) Gwin & Ferris, 2012a	*N* = 8 (7 m, 1f, 21–31 y)	Isometric right knee Ext & Flex (high & low effort); isotonic knee Ext (high & low effort)	Mean ERD	Frontal, central, parietal and occipital cluster cluster	Significant higher ERD in high effort [results not separated in knee or ankle contractions]
(60) Ismail et al., 2022	*N* = 12f (28 ± 6 y)	Isometric both Elbows Flex (5 exertion level extremely light to extremely hard)	Connectivity: Global & Local Graph Measures	84 ROIs	Activation of brain regions is sensitive for force level
(61) Ismail & Karwowski, 2023	*N* = 12f (∼)	Isometric both Elbows Flex (5 exertion level extremely light to extremely hard)	alpha & beta CSDs	84 ROIs	Efficiency in alpha and beta network is affected by exertion levels
(62) Moree et al., 2012	*N* = 21 m (27 ± 7)	Elbow flexion of 20 and 35% MVC	MRCP amplitude	C3/4, Cz, Fz, Pz	Significant difference between heavy and light weight in Readiness potential (Fz), weight raising (Fz, Cz), weight lowering (Cz)
(63) Siemionow et al., 2000	*N* = 8 (6 m, 2f; 32.1 ± 11.7)	Isometric right Elbow Flex (10, 35, 60, 85% MVC)	MRCP amplitude	Cz, C3	MRCP amplitude increases with joint forces, significance in every two adjacent force levels (C3 & Cz)
	*N* = 8 (6 m, 2f; 32.1 ± 11.7)	Isometric right Elbow Flex (35% MVC at slow, intermediate and fast rates of force developement	MRCP amplitude	Cz, C3	MRCP amplitude increases significant between every two adjacent rates (C3 & Cz)

BB, Biceps Brachii; BR, Brachialis; CCD, cortical current density; CMC, cortico muscular coherence; CSDs, current source densities; ED, endurance-trained; ERD, event-related desynchronization; ESP, EEG spectral power; Ext, extension; f, female; Flex, flexion; m, male; M1, primary motor cortex; MRCP, movement-related cortical potential; MVC, maximal voluntary contraction; MVT, maximum voluntary torque; PMC, premotor cortex; rMVC, relative MVC; ROIs, regions of interrest; S1, primary somatosensory cortex; SAC, supplementary motor area complex; ST, strength-trained; TB, Triceps brachii; TRSP, task-related spectral power; ∼, no information provided.

**Table 2:** Electrocortical correlates and type of muscle contraction of the included studies.

Author	Population	Muscle/exercise	EEG parameter	Brain area	Cortical findings
(64) Fang et al., 2001	*N* = 8 (6 m, 2f; 27.75 ± 7.21 y)	Left elbow Flex (Con & Ecc)	MRCP components	Cz, C3, C4, Fz	In all locations increased mean & peak NP, longer NP onset time and mean & peak PP (except Fz) in Ecc compared to Con
(65) Fang et al., 2004	*N* = 8 m (29.13 ± 2.36 y)	Left elbow Flex (Con & Ecc)	MRCP components	C3, C4, C6, F4, FC4, FC6	Differences Temporal and Spatial distribution of cortical activation patterns in eccentric and concentric movements
(46) Gwin & Ferris, 2012a	*N* = 8 (7 m, 1f, 21–31 y)	Right knee Ext & Flex (isotonic & isometric)	ERSP	Frontal, central, parietal and occipital cluster	CMC between the motor cortex and the lower limb. Gamma CMC is more prominent in isotonic contractions, beta-range oscillations more common in isometric contractions.
(66) Gwin & Ferris, 2012b	*N* = 8 (7 m, 1f, 21–31 y)	Right knee Ext & Flex (isotonic & isometric)	CMC in α, β and γ	Frontal, central, and parietal cluster	Different Patterns of Cortical Distribution in Gamma and Beta Coherence in Isometric and Isotonic Contraction Significant Beta and Gamma CMC between the contralateral motor cortex for all exercises
(67) Liu et al., 2019	*N* = 10 m (21–27 y)	Right elbow Flex and Ext [isometric & isokinetic (60°/s)]	¯XG CMC β & γ	C1, C5	¯XG of γ-CMC higher during isokinetic vs. isometric condition No significant differences in ¯XG of β-CMC between isokinetic and isometric condition
(68) Park et al., 2018	*N* = 16 (12 m, 4f, 22.43 ± 2.15 y)	Left elbow Flex [Con & ECC isokinetic (30°/s)]	ERD amplitude & onset (8–13-Hz)	C3 & C4	ERD onset times were significantly earlier in eccentric than concentric condition No significant difference in ERD amplitude in eccentric and concentric condition
(69) Shibata et al., 1997	*N* = 10 m (29.6 ± 9.2 y)	Right elbow flexion (brief phasic isometric & constant isometric)	mean amplitude MP & AM	Cz, C3 & C4	No significant differences in MP between dynamic and isometric condition Significant greater AM in isometric compared to dynamic condition

¯XG, grand average; AM, MRCP after movement; CMC, corticomuscular coherence; Con, concentric; Ecc, eccentric; ERD, event-related desynchronization; ERSP, event-related spectral perturbation; f, female; m, male; MP, motor potential; MRCP, movement-related cortical potential; NP, negative potential; PP, positive potential.

**Table 3:** Electrocortical correlates and volume of resistance exercise of the included studies.

Author	Population	Muscle/exercise	EEG parameter	Brain area	Cortical findings
(56) Flanagan et al., 2012	*N* = 7 ST m (22 ± 3 y)	Squat (PWR, FOR, VOL protocols)	MRA	Motor & sensory ROI	Significant differences in MRA in the last set VOL > PWR > FOR protocol in motor brain areas Significant differences in MRA in the last set VOL > PWR & FOR l in sensory brain areas

FOR, force; m, male; MRA, mean rectified amplitude; PWR, power; ROI, region of interest; ST, strength-trained; VOL, volume.

**Table 4:** Description of resistance exercise stimuli with the mechano-biological descriptors of resistance exercise (from Toigo & Boutellier, 2006).

	Type	Load	Reps (*n*)	Sets (*n*)	Rest sets	Frequency	Time	Time per mode and Rep	Rest Reps	TuT	Failure	RoM	Rec Session	Pred. Form
(57) Abeln et al., 2013	Knee extension	20% MVC	1	1	3 min		1 day	20 s isometric		20 s	No	0		Yes
		40% MVC	1	1	3 min			20 s isometric		20 s	No	0		Yes
		60% MVC	1	1	3 min			20 s isometric		20 s	No	0		Yes
		80% MVC	1	1	3 min			20 s isometric		20 s	No	0		Yes
		100% MVC	3	1	3 min			[]	1 min	[]	No	0		Yes
(50) Bayram et al., 2015	Elbow flexion	20% MVC	3	1	[]		1 day	10 s isometric	[]	30 s	No	0		No
		50% MVC	3	1	[]			10 s isometric	[]	30 s	No	0		No
		80% MVC	3	1	[]			10 s isometric	[]	30 s	No	0		No
		100% MVC	5	1	[]			∼8 s isometric	45 s	∼40 s	No	0		No
(58) Bayram et al., 2023	Elbow flexion	20% MVC	3	1	45 s		1 day	10 s isometric	∼45 s	30 s	No	0		No
		50% MVC	3	1	45 s			10 s isometric	∼45 s	30 s	No	0		No
		80% MVC	3	1	45 s			10 s isometric	∼45 s	30 s	No	0		No
		100% MVC	5	1	45 s			∼5 s isometric	45 s	∼5	No	0		No
(51) Cremoux et al., 2013	Elbow extension	25, 50, 75% rMVC	3	7	3 min		1 day	6 s isometric flexion, 6 s rest, 6 s isometric extension of each contraction level	6 s	756	No	0		Yes
(54) Dal Maso et al., 2012	Knee extension & flexion	20, 40, 60, 80% rMVC	2	10	3 min		1 day	6 s isometric flexion, 6 s rest, 6 s isometric extension of each contraction level	6 s	960	No	0		Yes
(55) Dal Maso et al., 2017	Knee extension & flexion	20, 40, 60, 80% rMVC	2	10	3 min		1 day	6 s isometric flexion, 6 s rest, 6 s isometric extension of each contraction level	6	960	No	0		Yes
(59) Fry et al., 2014	Knee extension	15, 30, 45, 60% MVT	3	5	2 min		1 day	∼5 s isometric	20, 30, 40, 50 s	300	No	0		Yes
(46) Gwin & Ferris, 2012a	Knee extension & flexion	9.1 kg	20	2	[]		1 day	∼1.5 s shorthening, ∼1.5 s Lenghtening	5 s	120	No	[]		No
		Mass of limb	20	2	[]			∼1.5 s shorthening, ∼1.5 s Lenghtening	5 s	120	No	[]		No
		100% SR	20	2	[]			∼3 s isometric	5 s	120	No	0		Yes
		25% SR	20	2	[]			∼3 s isometric	5 s	120	No	0		Yes
(60) Ismail et al., 2022	Elbow flexion	extremely light (RPE)	5	3	120 s		1 day	3 s isometric	30	45	No	0		Yes
		light (RPE)	5	3	120 s			3 s isometric	30	45	No	0		Yes
		somewhat hard (RPE)	5	3	120 s			3 s isometric	30	45	No	0		Yes
		hard (RPE)	5	3	120 s			3 s isometric	30	45	No	0		Yes
		extremely hard (RPE)	5	3	120 s			3 s isometric	30	45	No	0		Yes
(61) Ismail & Karwowski, 2023	Elbow flexion	extremely light (RPE)	5	3	120 s		1 day	3 s isometric	30	45	No	0		Yes
		light (RPE)	5	3	120 s			3 s isometric	30	45	No	0		Yes
		somewhat hard (RPE)	5	3	120 s			3 s isometric	30	45	No	0		Yes
		hard (RPE)	5	3	120 s			3 s isometric	30	45	No	0		Yes
		extremely hard (RPE)	5	3	120 s			3 s isometric	30	45	No	0		Yes
(62) Moree et al., 2012	Elbow flexion	20% 1RM	10	5	20 s		1 day	1 s shorthening, 1 s lengthening	6	100	No	∼126°		Yes
		35% 1RM	10	5	20 s			1 s shorthening, 1 s lengthening	6	100	No	∼126°		Yes
(63) Siemionow et al., 2000	Elbow flexion	10% MVC	35–40	1	5 min	1–2 per week	2 days	[]s isometric	∼5 s	[]	No	0	>= 3 days	Yes
		35% MVC	35–40	1	5 min			[]s isometric	∼5 s	[]	No	0		Yes
		60% MVC	35–40	1	5 min			[]s isometric	∼5 s	[]	No	0		Yes
		85% MVC	35–40	1	5 min	1–2 per week	2 days	[]s isometric	∼5 s	[]	No	0		Yes
		35% MVC	35–40	1	5 min			[]s isometric	∼5 s	[]	No	0	>= 3 days	Yes
		35% MVC	35–40	1	5 min			[]s isometric	∼5 s	[]	No	0		Yes
		35% MVC	35–40	1	5 min			[]s isometric	∼5 s	[]	No	0		Yes
(64) Fang et al., 2001	Elbow flexion	10% body weigth	50	1	5 min		1 day	∼5 s isometric,∼1 s shorthening	∼10 s	300	No	30		Yes
		10% body weigth	50	1	5 min			∼5 s isometric, ∼1 s lengthening	∼10 s	300	No	30		Yes
(65) Fang et al., 2004	Elbow flexion	100% MVC	40	1	0		1 day	1 s shorthening, 1 s lengthening	∼10 s	80	No	30		Yes
(66) Gwin & Ferris, 2012b	Knee extension & flexion	9.1 kg	20	2	[]		1 day	∼1.5 s shorthening, ∼1.5 s lenghtening	5 s	120	No	[]		No
		mass of limb	20	2	[]			∼1.5 s shorthening, ∼1.5 s lenghtening	5 s	120	No	[]		No
		100% SR	20	2	[]			∼3 s isometric	5 s	120	No	0		Yes
		25% SR	20	2	[]			∼3 s isometric	5 s	120	No	0		Yes
(67) Liu et al., 2019	elbow flexion & extension	30% MVC	40	1	[]		1 day	∼2 s shorthening, ∼2 s shorthening,	5 s	160	No	125		Yes
		30% MVC	40	1	[]			∼2 s isometric flexion ∼2 s isometric extension	5 s	160	No	0		Yes
(68) Park et al., 2018	Elbow flexion	30°/s	30	3	10 min		1 day	∼2 s shorthening	∼13 s	180	No	60		Yes
		30°/s	30	3	10 min			∼2 s lengthening	∼13 s	180	No	60		Yes
(69) Shibata et al., 1997	Elbow Flexion	20% MVC	5	10	30 s		1 day	2 s isometric flexion	10 s	100	No	0		Yes
		20% MVC	5	10	30 s			Brief isometric flexion	10 s	[]	No	0		Yes
(56) Flanagan et al., 2012	Squats	30% 1RM	3	3	3 min	1 per week	4 weeks	[]s lenghtening, []s shorthening	0	[]	No			
		95% 1RM	3	3	3 min			[]s lenghtening, []s shorthening	0	[]	No			
		80% 1RM	10	10	3 min			[]s lenghtening, []s shorthening	0	[]	No			
		6.8 kg	1	1				20 s isometric		20	No			

MVC, maximum voluntary contraction; rMVC, relative maximum voluntary contraction; MVT, maximum voluntary torque; Pred. Form, predefined exercise form; Rec, recovery; RoM, range of motion; RPE, rated perceived exertion; SR, subjective rating; TuT, time under tension; 1RM, one-rep maximum; [], missing information.

**Table 5:** Description of EEG methodology of included studies.

	Electrode configuration	Sampling rate	Filter processes	Artifact removal method	Source localization	EEG metrics
(57) Abeln et al., 2013	32 active electrodes	500 Hz	High-pass: 3.5 Hz Low-pass: 70 Hz Notch-filter: 50 Hz	• Channel neighbor interpolation • ICA • automatic artifact rejection (gradient criteria: < 50 μV/ms, amplitude criteria −200 to 200 μV, interval length: 200 m) • manual artifact rejection • Baseline Correction	sLORETA	Cortical Current Density
(50) Bayram et al., 2015	128 passive electrodes	250 Hz	High-pass: 0.1 Hz Low-Pass: 100 Hz	• manual artifact rejection	No	CMC
(58) Bayram et al., 2023	128 passive electrodes	250 Hz	High-pass: 0.1 Hz Low-Pass: 100 Hz	• Automatic artifact rejection (built in function BESA EEG analysis software) • reref common average	No	absolute and relative ESP
(51) Cremoux et al., 2013	64 active electrodes	[]	High-pass: 0.5 Hz	• ICA	No	20 Hz ERD
(54) Dal Maso et al., 2012	64 active electrodes	1,024 Hz	High-Pass: 0.5 Hz	• ICA	No	TRSP
(55) Dal Maso et al., 2017	64 active electrodes	1,024 Hz	High-Pass: 3 Hz Low-Pass: 100 Hz Notch-filter: 45–55 Hz	• re-reference to common average	No	CMC 13–21 & 21–31 Hz
(59) Fry et al., 2014	32 active electrodes	500 Hz	High-Pass: 0.5 Hz Low-Pass: 50	• semi-automatic artifact rejection (gradient criteria: < 50 μV, amplitude criteria −100 to 100 μV)	LORETA	Cortical Current Density
(46) Gwin & Ferris, 2012a	264 active electrodes	512 Hz	High-Pass: 1 Hz	• statistical criteria channel rejection [std ≥ 1,000 μV, kurtosis > 3 std, uncorrelated (*r* ≤ 0.4) with nearby channel (>0.1% time-samples)] • re-reference to common average • AMICA	Equivalent current dipole model	mean ERD
(60) Ismail et al., 2022	64 active electrodes	500 Hz	High-Pass: 1 Hz Low-Pass: 50 Hz	• automatic channel rejection (built in function EEGLAB “clean_raw data’) • ASR • AMICA	eLORETA	Connectivity: Global & Local Graph Measures
(61) Ismail & Karwowski, 2023	64 active electrodes	500 Hz	High-Pass: 1 Hz Low-Pass: 50 Hz	• automatic channel rejection (built in function EEGLAB ‘clean_raw data’) • common average re-referencing • ASR • AMICA	eLORETA	alpha & beta CSDs
(62) Moree et al., 2012	64 passive electrodes	100 Hz	High-Pass: 5 Hz Low-Pass: 40 Hz	• ICA • common average re-referencing • Manual trial rejection • baseline correction	No	MRCP amplitude
(63) Siemionow et al., 2000	2 [] electrodes	100 Hz		• manual artifact rejection	No	MRCP amplitude
(64) Fang et al., 2001	4 active electrodes	200 Hz	Low-Pass: 100 Hz	• manual artifact rejection	No	MRCP components
(65) Fang et al., 2004	64 active electrodes	250 Hz	Low-Pass: 50 Hz	• manual artifact rejection • baseline correction (−2 to −1.5 s)	No	MRCP components
(66) Gwin & Ferris, 2012b	256 active electrodes	512 Hz	High-Pass: 1 Hz	• statistical criteria channel rejection [std ≥ 1,000 μV, kurtosis > 3 std, uncorrelated (*r* ≤ 0.4) with nearby channel (>0.1% time-samples)] • re-reference to common average • AMICA	Equivalent current dipole mode	CMC in α, β and γ
(67) Liu et al., 2019	32 active electrodes	500 Hz	High-Pass: 0.5 Low-Pass: 40 Hz	• ICA	No	¯XG CMC β & γ
(68) Park et al., 2018	32 active electrodes	[]	High-Pass: 0.03 Low-Pass: 100 Hz Notch-filter: 60 Hz	• ICA	No	ERD amplitude & onset (8–13-Hz)
(69) Shibata et al., 1997	3 [] electrodes	1,000 Hz	High-Pass: 0.08 Hz Low-Pass: 30 Hz		No	mean amplitude MP & AM
(56) Flanagan et al., 2012	40 passive electrodes	100 Hz	Low-Pass: 50 Hz	• manual artifact rejection • spatial filter to remove Artifacts (eye and facial muscle)	No	MRA

AM, MRCP after movement; AMICA, adaptive mixture independent component analysis; ASR, artifact substance reconstruction; CCD, CMC, corticomuscular coherence; CSDs, current source densities; eLORETA, exact low-resolution brain electromagnetic tomography; ERD, event-related desynchronization; ESP, EEG spectral power; ICA, independent component analysis; LORETA, low-resolution brain electromagnetic tomography; MP, motor potential; MRA, mean rectified amplitude; MRCP, movement-related cortical potential; sLORETA, standardized low-resolution brain electromagnetic tomography; TRSP, task-related spectral power; XG, grand average; [], missing information.

**Table 6:** Allocation of brain localizations in studies utilizing channel-based brain localization methods to investigate intensity, type of contraction and volume of resistance exercise. Each study is identified by specific labeling numbers listed in Tables 1–3.

	Intensity	Type of muscle contraction	Volume
Frontal		(64)	(56)
Fontocentral	(50, 51, 54, 55, 63)	(64, 65, 67–69)	(56)
Centroparietal	(58)		(56)
Occipitoparietal			
Temporal			

**Table 7:** Allocation of brain localizations in studies utilizing source-based brain localization methods to investigate intensity, type of contraction and volume of resistance exercise. Each study is identified by specific labeling numbers listed in Tables 1–3.

	Intensity	Type of muscle contraction	Volume
Frontal	(46)	(46)	
Fontocentral	(46, 57, 59)	(46)	
Centroparietal	(46, 57, 59)	(46)	
Occipitoparietal	(46)	(46)	
Temporal			

### 3.1 Load of resistance exercises and electrocortical activity

Twelve of the included studies examined the load of RE and its underlying electrocortical activity. A summary of these studies is provided in Table 1. All twelve studies investigated either knee or elbow exertion at different RE loads. The exercise load was manipulated through external or internal factors. Most of the included studies adjusted the resistance load externally via the (relative) percentage of maximum voluntary contraction (50, 51, 54–59, 62, 63, 65, 67, 69) or rates of force development (63). Some studies controlled the different load levels internally using subjective ratings of exertion during isometric contractions (46, 60, 61). In terms of brain activity, four of the included studies investigated multiple sites of cortical areas (46, 57, 60, 61). The remaining studies primarily focused on the central motor areas of the brain, with most deriving cortical activity from a single or pair of electrodes (51, 54, 55, 63). The majority of the studies investigating load used EEG outcome parameters depicting changes in the frequency domain. While two studies focus on movement-related cortical potential (MRCP) as a parameter of the time domain (62, 63). Each of the studies showed a relationship between the load and changes in cortical activity. These changes in brain activity during RE are shown in increased cortical activity with muscle contraction (46, 51, 54, 57, 59, 62, 63). Decreased mean event-related desynchronization (ERD) in low-effort compared to high-effort tasks were found in isometric as well as isotonic RE (46). The highest cortical activity during RE was found to be in the primary motor cortex during RE (57). Furthermore, sensitivity and efficiency alterations in brain regions due to force level have been shown (61). Significant increases were observed in beta and low gamma brain activity from 50% to 80% of MVC during isometric elbow flexion (58). However, no significant differences were detected between 20%, 50%, and 80% MVC in delta, theta, alpha, beta, and low-gamma absolute and relative EEG spectral power. In contrast, Fry et al. (59), revealed gamma band cortical activity increased with the force of isometric knee extension, while other specific frequency bands remained unaffected by the resistance load. A significant effect of torque level is shown in movement preparation (62, 63) as well as during movement execution (62) in different RE loads of elbow movement derived from MRCPs.

### 3.2 Type of muscle contraction during resistance exercises and electrocortical activity

Seven of the included studies examined the type of muscle contraction and its electrocortical activities during RE (Table 2). All seven studies investigated either knee or elbow exertion including concentric, eccentric and isometric contractions. Besides extension or flexion, the RE performed differed in isotonic, isokinetic, or self-paced control of movement. Two studies examined electrocortical activity across multiple sites of cortical areas (46, 66), while five others used single electrodes focusing on central motor areas (64, 65, 67–69). Four of the studies allocated to the type of muscle contraction used event-related EEG outcome parameters, and three studies investigated CMC.

All seven studies showed that the type of muscle contraction significantly influences brain activity. These studies indicate differences in the spatial and temporal distribution of cortical activation according to the type of muscle contraction. It was found that gamma-range coherence was higher during isokinetic movements vs. isometric exercises (67). Patterns in CMC between the contralateral motor cortex and agonist lower limb muscle activity are shown corresponding to the type of muscle contraction. CMC in the gamma frequency band (31–45 Hz) is more pronounced during isotonic contractions, while beta-range (13–30 Hz) oscillations are more evident during isometric contractions (66). Differences in the time and the frequency domain of cortical activity in relationship to the type of muscle contraction during RE are shown by Park et al., 2018 (68) reporting an earlier desynchronization in 8–13 Hz frequencies in eccentric movement compared to concentric movement. Additionally in the time-domain, higher MRCP amplitudes and earlier onset times were associated with eccentric movements compared to concentric ones (64, 65). In comparison of isotonic and isometric contractions, isotonic movements exhibit alpha and beta ERD throughout the entire RE, whereas isometric contractions only cause alpha and beta ERD at the beginning and end of the contraction (46).

### 3.3 Volume of resistance exercise and electrocortical activity

A single study investigated the relationship between the volume of RE and electrocortical activity (Table 3). Cortical activity was recorded during different squatting exercise regimes, which varied in the number of repetitions and loads. Flanagan et al. 2012 (56) found an increase in the mean rectified amplitude in both sensory and motor areas of brain activity of highly strength-trained participants, depending on the exercise volume.

Specifically, the mean rectified amplitude during high-volume RE was significantly higher in both sensory and motor areas compared to high-force, power, and control modalities. Moreover, sensorimotor activity during high-volume exercise was significantly higher from the second to the sixth (last) set of exercises, in contrast to all other RE protocols (56).

### 3.4 Description of resistance exercise stimuli

Table 4 provides an overview of the utilized RE protocols in the included studies. Load magnitude was primarily defined as a percentage of maximum voluntary contraction (MVC) or relative MVC ranging from 15% to 100%. Some studies using fixed absolute loads (46, 66) or body weight percentages (64). Repetitions per set ranged from 1 to 50, with 1 to 10 sets per exercise. Rest intervals between sets varied from 30 s to 5 min. Most studies utilized isometric contractions lasting 2–3 s, while dynamic movements equally incorporated shortening and lengthening phases of 1–2 s each. Time under tension (TUT) ranged from 20 to 960 s, with some studies implementing varying load levels within sets (51, 54, 55, 59). Considering the different conditions of resistance levels, the TUT is calculated for the different load levels tested in the studies per condition is 75 (59), 240 (54, 55) and 252 (51) seconds. Rest periods between repetitions for submaximal contractions ranged from 5 to 50 s, while maximal contractions had 45–60 s rest periods. Range of motion was reported for elbow movements (30°–126°) but is not reported in dynamic lower body exercises (46, 56, 66). Notably, no studies aimed for volitional muscular failure. While most studies provided clear anatomical definitions of exercises, some lacked detailed descriptions of movement ranges (46, 66) or postures during isometric contractions (50, 58).

### 3.5 Description of methodological details using EEG

Table 5 provides an overview of methodological details of the included studies regarding EEG. Variability is shown in the electrode configurations and sampling rates. The number of electrodes ranges from 2 to 264, with a mix of active and passive types, while sampling rates vary between 100 and 1,024 Hz. Multiple studies use filters in the preprocessing of EEG data, with low-pass filter cutoffs ranging from 30 Hz to 100 Hz and high-pass filter cutoffs around 0.5 Hz to 1 Hz. Moreover, some studies used notch filters to remove specific frequencies (50 Hz or 60 Hz). Vast heterogeneity within the sample of investigated studies appears in the further preprocessing, artifact rejection methods, and EEG metrics. While independent component analysis (ICA) is commonly used, artifacts are also removed based on visual manual inspection, semi-automatic, and automatic artifact rejection using Artifact Subspace Reconstruction (ASR). Further, some studies implement statistical criteria to remove channels or data, while other studies implement additional steps like re-referencing or baseline correction.

The reported EEG metrics (Table 5) show a variety of outcome parameters used to measure electrocortical activity during RE. Most of the included studies utilized time-domain parameters to examining neural activity over time and quantifies the brain activity to specific events (71). Moreover, frequency parameters are frequently used to show how different frequencies contribute to the overall brain activity (71). One study used Event-Related Spectral Perturbations (ERSPs) to analyze time and frequency domain (46), which facilitates the examination of how different frequencies change over time (72). Additionally, some studies used connectivity measures to investigate functional relationships between different brain regions (60, 61) or between the brain and muscles (50, 55, 66, 67).

Most of the studies focused on channel-based brain locations and examined fronto-central brain activation (Table 6). Only three studies used source-based localization to investigate cortical activity at varying load levels (46, 57, 59) and a single examines the type of muscle contraction and underlying cortical activation (46). These focused on fronto-central and centroparietal brain areas associated with sensorimotor processing (Table 7).

### 3.6 Quality assessment

**Figure 2:**
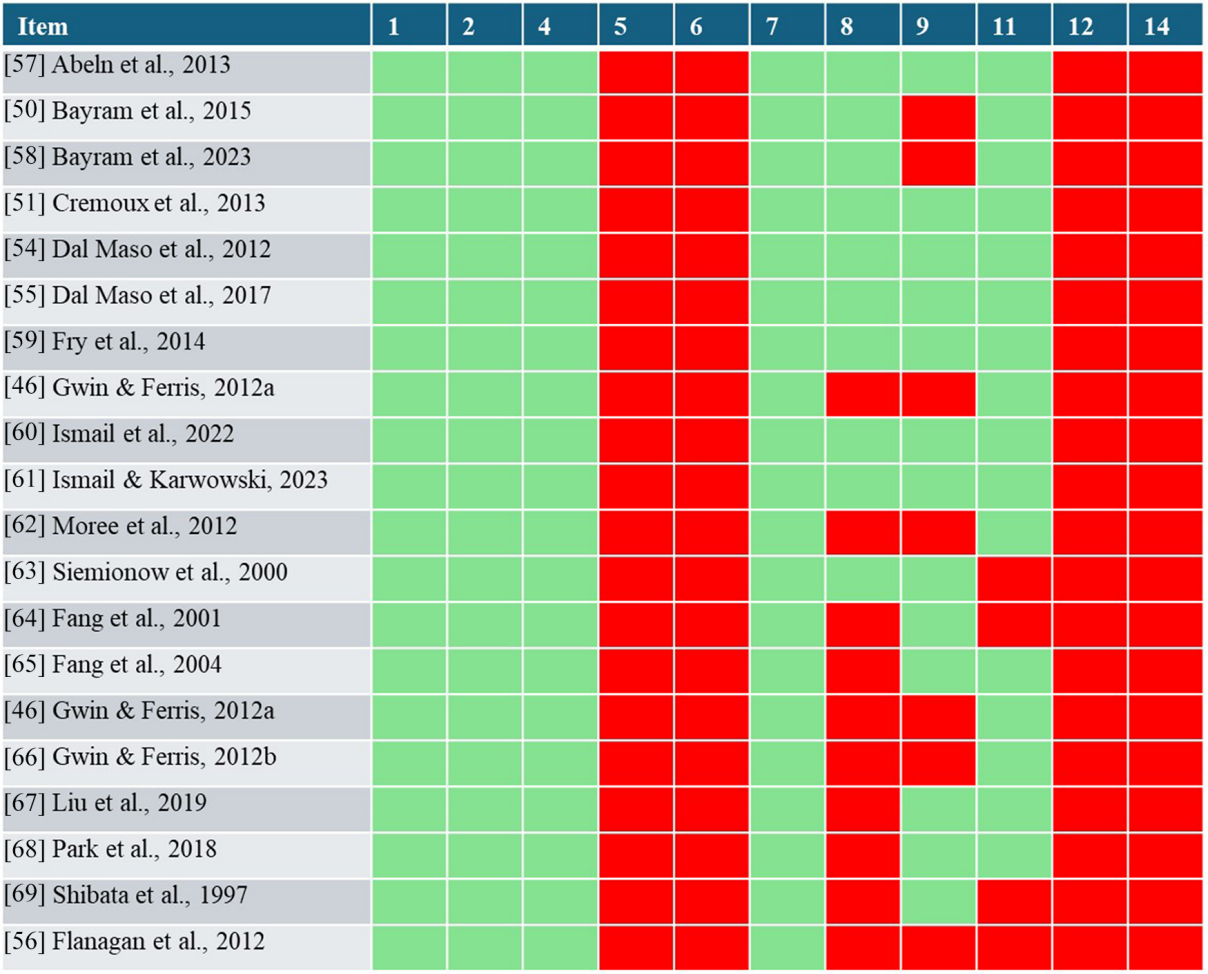
Results of the NHLBI quality assessment. With red indicating a potential risk of bias and green indicating minimized bias risks for each item and study.

Figure 2 reports the results of the NHLBI quality assessment. The ratings of the studies highly varied depending on the items. All included studies demonstrated an increased risk of bias regarding items 5, 6, 12, and 14. In contrast, items 1, 2, 4, and 7 indicated a low risk of bias in all studies. The assessment of studies revealed potential sources of bias in both RE protocols and cortical activity measurements. Regarding item 8, most studies examined multiple load levels, facilitating dose-response relationship understanding. However, studies using only two resistance levels (46, 62) or varying both volume and exercise load potentially confound the insights into this relationship (56). Additionally, studies investigating different types of muscle contractions were rated as having a potential risk of bias due to the difficulty in quantifying contraction types (64–69). Item 9 and 11 assesses whether the exposure measures (resistance exercise) and outcome measures (cortical activity) are clearly defined, valid, reliable and consistently performed. The lack of anatomical definition in the description of RE stimuli (Table 4) reveals missing information on range of motion (46, 56, 66) or joint angle (50, 58), potentially biasing the use of RE. In terms of cortical activity measurement (Item 11), potential risks were identified in some studies due to a low number of electrodes (≤4), non-standardized montages (63, 64, 69), incomplete EEG system information (69), or high data noise risk (56).

## 4 Discussion

This review aims to provide an overview of electrocortical activity underlying RE concerning the variables load, type of muscle contraction, and volume of exercise. The key findings suggest that RE stimulates the activation of cortical brain areas. These activations show a clear association between the type of muscle contraction and the load prescribed during RE. The electrocortical correlates also suggest a potential association with exercise volume. This suggests that electrocortical activity is moderated by the prescribed RE stimuli. Methodologically, the studies differed substantially with regards to the investigated brain sites and the analytical approaches chosen to analyze brain activity. Regarding EEG outcome parameters, the studies primarily focused on event-related changes as well as alterations in the frequency and topography of cortical activation. The fronto-cental brain area seems to be the primary focus in current EEG research during RE.

### 4.1 Load of resistance exercise and electrocortical activity

In summary, the present review suggests that the load of RE affects cortical activations. Associations between exercise load and cortical activity have also been demonstrated for endurance exercise (73, 74). Similar to endurance exercise, the manipulation of RE load has been linked to specific modulations of cortical activity. It affects the activation of sensorimotor areas, particularly in the specific frequencies of Theta, Alpha and, Beta (73, 74).

Due to the high temporal resolution of the EEG, changes in cortical activation have also been associated with the specific phases of movement planning and execution (62, 63). Increased load levels are associated with a rise in descending cortical signals, indicated by a higher amount (50, 57) and efficiency (60) of local brain activation during force production. A possible underlying mechanism is that higher force demands result in a larger number of active motoneurons and higher discharge rates of motoneurons (75). This observation aligns with prior studies investigating finger or hand grip exercises (76, 77), displaying increasing neural demands when force levels increase.

Further, frequency-specific changes in cortical oscillations specific to exercise intensity have also been demonstrated in finger-contraction tasks (78). The present review corroborates this finding, indicating that higher loads during knee and elbow exertion are associated with frequency-specific changes in cortical activation (54, 58, 59, 61). While low gamma has been associated with visual feedback processing and overall increased attention or arousal when performing contraction, the direct relationship with force level is not clear (58, 59). In contrast, alpha and beta have been associated with motor planning, sensorimotor processing, and sensory integration (54, 58, 61). Increased beta activation with higher force demands is supported by previous literature on isometric finger contraction (78) and is associated with central activation of motoneurons (77). However, these beta changes are potentially influenced by the age of the population (58).

In summary, the load of RE affects cortical activations, with specific modulations in sensorimotor areas and frequency-specific changes in cortical oscillations. This aligns with previous literature on endurance exercise workloads and emphasizes the role of RE load in neural demands.

### 4.2 Type of muscle contraction in resistance exercise and electrocortical activity

Next to the RE load, the present review revealed that different types of contractions modulate the cortical activations underlying RE. When comparing eccentric and concentric contractions, temporal and frequency-specific differences in cortical activation emerge (64, 65, 68). These changes have been observed in preparation, execution, and feedback processing phases of movement (64, 65, 68). This is supported by shifts in onset timing and amplitude variations in MRCP components depending on concentric or eccentric elbow exertion (64, 65, 68). Greater effort and more extensive neural networks seem to be associated with the control of eccentric compared to concentric movements (64, 65). Consequently, eccentric contractions, which might be associated with unique control strategies and increased difficulty (64), might require more brain resources for the preparation due to the unfamiliarity of eccentric RE (68). These differences in contraction types are attributed to variations in the requirements of somatosensory activity and feedback processing (68, 69). The heightened Mu ERD is thought to be associated with increased excitability in the thalamocortical feedback loop of somatosensory processing (68). Shibata et al. (1997) emphasized that the timeframe following movement onset in MRCP largely represents the somatosensory response to RE.

Similar to the comparison of eccentric and concentric movement differences in sensory processing were depicted through the comparison of static and dynamic contractions. Differences in frequency-specific coherences appeared between dynamic and static contractions (66, 67). Both studies suggest that dynamic movements are associated with increased gamma coherence, while isometric contractions are associated with increased beta coherence. The shift towards increased CMC in gamma frequencies during dynamic movements is associated with an increased amount of proprioception (66, 67). Gwin and Ferris (2012a) support this observation, showing time- and frequency-specific differences during isotonic and isometric contractions (46). Alpha and beta ERD throughout resistive isotonic movements are associated with sensorimotor processing. In contrast, during isometric contraction, muscle shortening and tendon lengthening occur especially at the onset and offset of the RE. Thus, the amplitude of desynchronization in the alpha and beta bands is lower during the holding phase of the isometric contraction compared to the dynamic isotonic contraction (79, 80). Moreover, the distribution of activated brain areas may differ depending on the type of muscle contraction. Fang et al. (2004) suggest that the increased task complexity of eccentric movement is associated with a broader distribution of activated cortical areas (65).

In summary, different types of muscle contractions, such as eccentric and concentric contractions, as well as dynamic and static contractions, affect cortical activations with specific temporal and frequency-specific modulations. This highlights the potential impact of the type of muscle contraction on the requirements of somatosensory activity. These requirements may be evoked by unfamiliarity, difficulty, and complexity of contracting, possibly leading to differences in central neural effort and control strategies of contractions.

### 4.3 Volume of resistance exercise and electrocortical activity

While multiple studies investigated the effect of exercise load and type of muscle contraction on cortical activity underlying RE, the role of the volume of prescribed RE has been largely ignored and investigated by only one study, which linked the increase of RE volume to fatigue (56). Increased cortical activity related to fatigue has been observed in both sensory and motor brain areas. In the motor region, the change in brain activity may indicate an attempt to compensate for the loss of force production capabilities during repeated exercise (81, 82). In contrast, increased activity in sensory brain areas associated with the volume of RE may represent fatigue as a central indicator for acute peripheral feedback (83, 84).

These findings have been shown to be limited to high loads by highly trained athletes. Unlike other studies in this review, cortical activity was recorded during squatting, a complex multi-joint activity (85). Using the mean rectified amplitude as a continuous EEG outcome parameter, the study does not provide information on the effects of volume or fatigue at the event or frequency level. Despite the limited evidence regarding the impact of volume on cortical activity, this study emphasizes fatigue and exercise variables, especially exercise load, as essential factors for a better understanding of brain activity during RE.

### 4.4 Quality assessment

The quality assessment of included studies (Figure 2) highlighted that certain items were consistently rated as either “no” or “yes” across all reviewed studies. Although all studies reported the research question or objective of the study clearly, some studies failed to define specific hypotheses (46, 58–61, 63–65, 68, 69). Further, none of the studies performed a sample size justification or determined the statistical power. Thus, the revised studies can be primarily classified as exploratory and hypotheses-generating research.

All studies present potential bias due to the lack of blinding of the participants and personnel, and the lack of appropriate consideration for confounders in their statistical analyses. The potential risks of bias indicated by items 9 and 11 highlight the importance of clearly defining and reporting both the exercise regime (independent variable) and cortical outcome measure (dependent variable) to facilitate meaningful and comparable results across studies.

### 4.5 Analytical procedures in current EEG resistance exercise research

The current review presents significant challenges in the application of EEG during RE due to heterogeneity and inconsistencies across the studies in methodologies (electrode configurations, sampling rates, filtering techniques, artifact rejection methods, and EEG metrics; see Table 5) and the prevalence of movement and muscle artifacts. Muscle artifacts share frequencies power with brain activity in frequencies above 20 Hz (71), with gamma frequency (30–50 Hz) being especially vulnerable to muscle contamination (86). To address these issues, both practical and technical approaches have been developed to reduce artifacts in sport settings (87).

From a hardware perspective, using a sufficient number of active electrodes (≥35, preferably ≥64 for higher intensity movements (88, 89); and ensuring consistent electrode placement can support artifact removal based on the effective decomposition of brain- and non-brain contributions to the electrocortical sum signal (90). Post-recording signal processing techniques, such as high-pass filters with a cut-off frequency of 1.5–2 Hz for vigorous mobile experiments, are also recommended (89). Such filter may also reduce the impact of slow rhythmic movement, such as the concentric-eccentric movement phases, on the recorded brain signals. For artifact rejection, a combination of methods and parameter tuning is commonly advised (47, 86, 91, 92), with blind source separation techniques like ICA being particularly effective to differentiate cortical activation from noise (86, 93).

Due to the lack of ground truth in EEG data during physical activity (86, 93) and the unavoidable artifacts during RE (87, 89), there is no one-size-fits-all solution for EEG application and signal processing during RE. Future research should focus on developing application-specific solutions that consider the unique challenges posed by different types of RE (47, 86, 91, 92). Here, a multimodal approach combining EEG, electromyography, and dynamometer may allow for effective identification of non-brain components in the data. In general, it's crucial to use fixed criteria for artifact suppression to ensure objectivity. Additionally, all pre-processing steps should be adequately documented to facilitate reproducibility (94). The reported EEG metrics demonstrate heterogeneity of outcome parameters used to investigate electrocortical activity during RE. Nevertheless, the present review demonstrated that both the time- and the frequency-domains of the EEG signal were affected by different RE prescriptions. Therefore, future studies should take advantage of the EEG´s properties and consider the utilization of EEG parameters that take into account both aspects of the EEG signal, such as event-related spectral perturbations (46, 95).

### 4.6 Limitations

The current literature significantly highlights the impact of the RE variables load, type of muscle contraction and volume of RE on cortical activity underlying RE. Nevertheless, some limitations of the present review need to be highlighted.

Although this review only included healthy young participants, a difference in cortical activity has been shown between healthy endurance and strength-trained participants (54, 55). Furthermore, factors such as age, gender, expertise, and socio-economic factors can affect electrocortical activity (96). The current results should be applied with caution to other populations. Secondly, the current literature is inconsistent regarding approaches chosen to quantify electrocortical activity. Although all of the included studies investigated brain areas associated with sensorimotor processing, there is heterogeneity in the examined areas and in the methods used to localize brain activity. Beyond the primary motor cortex (97), multiple brain sites are shown to be involved in sensorimotor processing to perform RE (83). Some studies utilize source-based localization, while others use channel-based localization. Additionally, there is a lack of consistency in the EEG outcome parameters used across studies to investigate brain activity in RE. Thirdly, this review incorporates heterogeneous RE regimes and their underlying cortical processing. The varying methods used to modulate load during RE in this study highlight the absence of a standardized approach for controlling external and internal load during RE (18). The current heterogeneity in exercise stimulus and neurophysiological outcome measures limits the current evidence regarding electrocortical activity and the associated potential neuronal adaptations to RE (32, 98).

## 5 Conclusion

The present review identified the impact of the specific prescription of RE training variables on underlying cortical activity derived from EEG. In line with the current literature, this review underscores the limited and inconsistent evidence regarding the electrocortical activity during RE and confirms the need to address conceptual questions. To improve the investigation of electrocortical activity during RE in future studies, both technical and practical approaches should be considered by focusing on (a) the design and description of RE stimuli, and (b) the application of EEG during RE. Given the current lack of established indicators, EEG offers potential insights into the brain during RE. It potentially provides an indicator for neuromuscular involvement during RE to quantify internal load.

Future EEG-based studies focusing on brain function during RE should consider both the modulation of RE stimuli and methodological aspects specific to EEG in RE settings. The modulation of RE stimuli should be carefully designed and described, as proposed by Toigo & Boutellier (2006). Regarding methodological considerations for EEG in RE, the importance of spatial, temporal, and spectral characteristics in brain activation is highlighted, while simultaneously controlling for artifacts, especially from motion and muscle sources. Ultimately, new insights into the acute cortical mechanisms of RE could potentially optimize the control of RE stimuli for both training and rehabilitation purposes from a brain perspective.

## Data Availability

The original contributions presented in the study are included in the article/Supplementary Material, further inquiries can be directed to the corresponding author.
